# Fabrication of Bacterial Cellulose Nanofibers/Soy Protein Isolate Colloidal Particles for the Stabilization of High Internal Phase Pickering Emulsions by Anti-solvent Precipitation and Their Application in the Delivery of Curcumin

**DOI:** 10.3389/fnut.2021.734620

**Published:** 2021-09-07

**Authors:** Rui Shen, Dehui Lin, Zhe Liu, Honglei Zhai, Xingbin Yang

**Affiliations:** ^1^Shaanxi Engineering Laboratory for Food Green Processing and Safety Control, Shaanxi Key Laboratory for Hazard Factors Assessment in Processing and Storage of Agricultural Products, College of Food Engineering and Nutritional Science, Shaanxi Normal University, Xi'an, China; ^2^Department of Pediatrics, Children's Nutrition Research Center, Baylor College of Medicine, Houston, TX, United States

**Keywords:** BCNs/SPI colloidal particles, anti-solvent precipitation, high internal phase pickering emulsions, antioxidation, delivery of curcumin

## Abstract

In this study, the anti-solvent precipitation and a simple complex method were applied for the preparation of bacterial cellulose nanofiber/soy protein isolate (BCNs/SPI) colloidal particles. Fourier transform IR (FT-IR) showed that hydrogen bonds generated in BCNs/SPI colloidal particles *via* the anti-solvent precipitation were stronger than those generated in BCNs/SPI colloidal particles self-assembled by a simple complex method. Meanwhile, the crystallinity, thermal stability, and contact angle of BCNs/SPI colloidal particles *via* the anti-solvent precipitation show an improvement in comparison with those of BCNs/SPI colloidal particles *via* a simple complex method. BCNs/SPI colloidal particles *via* the anti-solvent precipitation showed enhanced gel viscoelasticity, which was confirmed by dynamic oscillatory measurements. Furthermore, high internal phase Pickering emulsions (HIPEs) were additionally stable due to their stabilization by BCNs/SPI colloidal particles *via* the anti-solvent precipitation. Since then, HIPEs stabilized by BCNs/SPI colloidal particles *via* the anti-solvent precipitation were used for the delivery of curcumin. The curcumin-loaded HIPEs showed a good encapsulation efficiency and high 2,2-diphenyl-1-picrylhydrazyl (DPPH) removal efficiency. Additionally, the bioaccessibility of curcumin was significantly increased to 30.54% after the encapsulation using the prepared HIPEs. Therefore, it can be concluded that the anti-solvent precipitation is an effective way to assemble the polysaccharide/protein complex particles for the stabilization of HIPEs, and the prepared stable HIPEs showed a potential application in the delivery of curcumin.

## Introduction

Emulsion is a dispersed heterogeneous system composed of two or more completely or partially immiscible liquids, which has been widely used in foods, pharmaceuticals, cosmetics, and other fields (1). Recently, high internal phase emulsions (HIPEs) belonging to the class of concentrated emulsions with more than 74% (v/v) of the internal phase have aroused much interest in the food industry because HIPEs offer a promising approach for converting liquid oil into solid. However, the stability of HIPEs is a critical challenge due to the usual occurrence of phase transitions and an increase in the volume of the internal fraction to a threshold. Traditional HIPEs are usually stabilized by the high dosages (10–50%) of surfactants or small-molecule emulsifiers, which were considered as a metastable system with thermodynamic instability (2, 3). Therefore, Pickering emulsions stabilized by solid particles have attracted great attention because of their outstanding stability, resulting from the irreversible adsorption of particles at an oil–water interface (4, 5). At present, inorganic particles, including silicon dioxide, titanium dioxide, and clay, are widely used as stabilizers for HIPEs due to their efficient emulsifying capacity (6, 7). However, the application of inorganic particles in the food industry is limited due to their toxicity risks (8). Therefore, it is necessary to explore food-grade stabilizers for HIPEs (9).

Recently, various food materials (such as proteins, polysaccharides, and lipids) have been widely used for the development of food-grade particles for Pickering emulsion (10–12). However, it has been demonstrated that the solid particles from proteins or polysaccharides alone display poor emulsification properties as these are used for the stabilization of HIPEs. Thus, chemical modifications are usually used to improve the emulsifying capacity of polysaccharide or protein particles while the chemical modification is unable to adapt to the development of the green food processing industry (13, 14). It has been reported that the complex particles of protein and polysaccharides by the self-assembly can stabilize the HIPE effectively. However, the self-assembly methods would largely affect the properties of complex particles because different self-assembly methods lead to different intermolecular interactions between the complex particles (e.g., the electrostatic interactions, hydrogen bonds, van der Waals' force, and steric hindrance) (15). Thus, it is necessary to investigate the effects of self-assembly methods on the properties of the complex particles.

In this work, bacterial cellulose nanofiber/soy protein isolate (BCNs/SPI) colloidal particles were prepared by using the anti-solvent precipitation and a simple complex method. Then, in this study, the properties of BCNs/SPI colloidal particles, including microstructure, thermostability, contact angle, and rheological behavior, were compared. Meanwhile, BCNs/SPI colloidal particles were used for the stabilization of the HIPEs, and their stability was studied. Afterward, the super stable HIPEs were used for the delivery of curcumin. The present study has investigated the delivery properties, including the antioxidant activity of curcumin, bioaccessibility of curcumin during *in vitro* digestion, and release of free fatty acids (FFAs).

## Materials and Methods

### Materials

Bacterial cellulose (BC) producing strain *Komagataeibacter hansenii* CGMCC 3917 was donated by the Fermentation Technology Innovation Laboratory of Northwest A & F University (Yanglin, Shaanxi, China). SPI (purity 95%), curcumin, and bile extract were purchased from Yuanye Bio-Technology Co., Ltd. (Shanghai, China). Sunflower seed oil was purchased from the local supermarket (Xi'an, China). Porcine mucin, porcine pepsin (≥300 U/mg), and porcine pancreatin (4,000 U/g) were obtained from Aladdin Bio-Technology Co., Ltd. (Shanghai, China), and other chemicals were of analytical grade.

### Sodium Dodecyl Sulfate-Polyacrylamide Gel Electrophoresis Measurements

Sodium dodecyl sulfate-polyacrylamide gel electrophoresis (SDS-PAGE) measurements were measured according to the method reported in a previous literature study (16). About 5.0% polyacrylamide stacking gels and 15.0% polyacrylamide running gels were prepared by using a gel kit. Then, the gels were stained with Coomassie Brilliant Blue. Next, the stained gels were faded by using destaining solution (glacial acetic acid: methyl alcohol: water = 10: 45: 45 v: v: v). Finally, the gel imager (Bio-Rad, Hercules, CA, USA) was used for performing scanning and analysis.

The protein composition of SPI was analyzed by using SDS-PAGE and mainly consists of 7S and 11S globulin (Supplementary Figure 1). Three electrophoretic bands were separated clearly as shown corresponding to the molecular weights of 72, 68, and 52 kDa, which were observed as the α', α, and β subunits of 7S globulin. Electrophoretic bands corresponding to the molecular weights of 35 and 20 kDa represent the acidic and basic peptides of 11S globulin, respectively.

### The Preparation of BCNs

Bacterial cellulose was produced according to the method in our previous study (17). Briefly, *K. hansenii* CGMCC 3917 was cultured at an aerobic environment (30°C) in a fermentation medium (pH 5.0) containing glucose 2% (w/v), yeast extract 0.5% (w/v), K_2_HPO_4_ 0.1% (w/v), MgSO_4_ 1.5% (w/v), and ethanol 2% (v/v). Cellulose membranes were harvested after the 14-day static cultivation. Then, the membranes were rinsed under running water overnight, followed by the treatment with 0.1 M NaOH solution at 80°C for 2 h. After that, the membranes were washed several times with deionized water until it was natural. Next, BC was hydrolyzed with 2.5 M HCl solution at 70°C for 1 h under magnetic stirring conditions (200 rpm). Afterward, the suspension was washed with deionized water and centrifuged at 10,000 *g* until the pH was neutral. Finally, BCNs suspension was obtained and stored in a fridge at 4°C for a successive study.

### Preparation of BCNs/SPI Complex Particles

Bacterial cellulose nanofiber/SPI complex particles were prepared by using the anti-solvent precipitation and a simple complex method. For the anti-solvent precipitation, SPI stock solution (1.25%, w/v) was obtained by dissolving an SPI powder in ethanol solution (70%, v/v) through stirring at room temperature (25°C). BCNs suspension with the concentration of 0.1% (w/v) was prepared by dissolving BCNs in deionized water. Then, SPI solution and BCNs solution were mixed in the ratio of 1:2.5 (v: v). Next, BCNs/SPI mixtures were prepared in the ratio of 1:5 (w/w) and sheared at 8,000 rpm for 4 min using a high-speed homogenizer (S10, SCIENTZ, Zhejiang, China). Finally, ethanol and excess water were removed by rotary evaporation and centrifugation, and BCNs/SPI colloidal particles with a concentration of 2% were obtained and marked as A-BCNs/SPI. For a simple complex method, SPI stock solution (1.25%, w/v) was obtained by dissolving a powder in deionized water through stirring at room temperature (25°C). BCNs suspension was prepared by dissolving these in the deionized water. Then, BCNs/SPI mixtures were prepared in the ratio of 1:5 (w/w) and sheared at 8,000 rpm for 4 min using a high-speed homogenizer (S10, SCIENTZ, Zhejiang, China). Finally, excess water was removed by centrifugation (4,000 rpm, 10 min), and BCNs/SPI colloidal particles with a concentration of 2% were obtained and marked as S-BCNs/SPI.

### Preparation of HIPEs

A-bacterial cellulose nanofiber/soy protein isolate colloidal particles and S-BCNs/SPI colloidal particles were used as a stabilizer to prepare HIPEs, which were marked as A-HIPEs and S-HIPEs, respectively. Oil in water (O/W) HIPEs were prepared with the oil fraction of 75% by one-step homogenization. Briefly, the continuous phase (BCNs/SPI complex particle solution 2%) and dispersed phase sunflower seed oil were homogenized at 25,000 rpm for 3 min using a high-speed homogenizer (S10, SCIENTZ, Zhejiang, China).

### The Loading of Curcumin

In this study, three types of systems were used for the loading of curcumin. Type I: curcumin was added into the oil with a concentration of 0.5 mg/ml (w/v), then the curcumin-loaded oil was obtained and marked as cur-oil. Type II: the HIPEs (oil fraction, 75%) stabilized by BCNs/SPI colloidal particles *via* the anti-solvent precipitation were used for the loading of curcumin. Briefly, the continuous phase (A-BCNs/SPI colloidal particle solution, 2%) and dispersed phase (curcumin-loaded oil) were homogenized at 25,000 rpm for 3 min, then the curcumin-loaded HIPEs were obtained and marked as cur-HIPEs. Type III: emulsions (oil fraction, 50%) stabilized by a surfactant (Tween 80 solution, 2%) were used for the loading of curcumin and set as a control group (18). Briefly, the continuous phase (Tween 80 solution, 2%) and dispersed phase (curcumin-loaded oil) were homogenized at 25,000 rpm for 3 min, then the curcumin-loaded emulsion stabilized by Tween 80 was obtained and marked as cur-TEs.

### Zeta-Potential Measurements

The particle charge [zeta- (ζ-) potential] of colloidal particles and HIPEs was performed using the nano-zeta potential analyzer (Malvern Instruments Ltd., Malvern, UK). Briefly, the samples were diluted to 0.01% (v/v), and 0.5 ml of diluted dispersion was injected into a measurement cell for measurement. AI and RI of samples were set as 0.010 and 1.590. Each experiment was measured three times.

### Particle Size

The particle size and polydispersity index of BCNs, SPI, and BCNs/SPI colloidal particles were measured by using a laser particle analyzer (Brookhaven, NY, USA). The samples were diluted 50-fold before the measurement. And each experiment was measured three times.

### Microstructures

The microstructure of BCNs/SPI complex particles was characterized using scanning electron microscopy (S-3400N, HITACHI, Tokyo, Japan) The freeze-dried samples were coated with gold by an E-1045 sputter coater (HITACHI, Tokyo, Japan). The images were collected with 10,000 × and 1,000 × magnification at an accelerating voltage of 10.0 kV (19).

The microstructures of HIPEs were observed using the Axio Imager Upright Microscope (ZEISS, Oberkochen, Germany). A drop of HIPE was put on the glass microscope slide, then the prepared samples were observed under the microscope (20).

### FT-IR Spectroscopy

The FTIR spectra of BCNs/SPI complex particles were recorded using a FT-IR spectrometer (BRUKER, Vertex70, Karlsruhe Germany) at 4,000–400 cm^−1^ with a resolution of 4 cm^−1^ (21).

### X-Ray Diffraction

X-ray diffraction (XRD) patterns were recorded using an x-ray diffractometer (Rigaku, Smart Lab, Tokyo, Japan) at 40 kV and 45 mA. The samples were scanned from 5 to 50° with a step of 0.02° 2θ intervals (4).

### Thermostability Analysis

The thermostability analysis of colloidal particles was characterized using the thermogravimetric analysis (TGA) and differential scanning calorimetry (DSC). Thermogravimetry (TG) was performed with 5 mg of nanoparticles under nitrogen atmosphere (N_2_ flow was 20 ml/min) using a Thermoanalyzer System (Q1000DSC + L NCS + FACS Q600SDT, TA Instrument, New Castle, DE, USA) according to the previous method. The samples were heated from 20 to 800°C for 10°C/min, and the derivative thermogravimetry (DTG) was obtained by using the derivation for the TGA curve (22).

Differential scanning calorimetry (Flash DSC1, Schwerzenbach, Switzerland) was carried out for thermal analysis. About 3 mg of freeze-dried samples were placed in an aluminum pan and measured at the rate of 10°C/min over the range of 20–250°C (23).

### Fluorescence Spectroscopy

The fluorescence intensity of samples was determined by using the previously reported method, with some modifications (24). A fluorescence spectrophotometer (F-7000, HITACHI, Tokyo, Japan) was used, the concentration of colloidal particle dispersions was fixed to 2 mg/ml. The emission spectra were recorded between 290 and 450 nm. The scanning parameters were set as follows: the excitation wavelength of 280 nm, the scanning speed of 100 nm/min, and the slit width of 10 nm for excitation and emission. All data were the average of three runs.

### Contact Angle

An optical contact angle measuring device (OCA20, Dataphiscis Instruments GmbH, Filderstadt, Germany) was used for the evaluation of the hydrophilic/hydrophobic characteristics of BCNs, SPI, and BCNs/SPI colloidal particles. In brief, the samples were spread on the tablets and dried at 37°C. Then the dried samples were placed in the equipment platform, 2 μl ultrapure water (pH 7.5) was successively deposited on the surface using a high-precision syringe. The water drop image was recorded *via* a high-speed video camera for a second. After that, the droplet profile data was fitted to the LaPlace–Young equation, and the contact angle was calculated according to the pervious description (25).

### Rheological Properties

Dynamic rheometer (ZX7M-AR1000, TA Instruments, New Castle, DE, USA) was used for the measurement of the rheological properties of BCNs/SPI colloidal particles and HIPEs (10). The linear viscoelastic domain of particles was determined *via* an oscillatory stress sweep at a fixed frequency (1 Hz) before carrying out the oscillatory measurements. Then, a storage modulus (G′) and loss modulus (G″) were measured from 0.1 to 1,000 Pa at a frequency of 1 Hz. A steady shear flow model was used for the measurement of the viscosity of particles. The viscosity (η) was recorded from 0.1 to 100 s^−1^ at 25°C under the condition of linear mode. For a thixotropic property analysis, the initial shear rate was set at 0.1 s^−1^ for 300 s. Then, the shear rate was increased to 10 s^−1^ for 300 s. Finally, the shear rate was recovered to 0.1 s^−1^ for 300 s, and the viscosity (η) was recorded.

### Stability of HIPEs

Long-term storage was performed for testing the stability of HIPEs. Briefly, HIPEs were sealed in a serum bottle for 1 month at room temperature. The visual appearance and droplet size distribution of HIPEs were monitored for 1 month. The particle size and size distribution of HIPEs were determined using a laser particle size analyzer (LS13320, Beckman, Indianapolis, IN, USA). HIPEs were sufficiently diluted by deionized water to avoid multiple scattering. The refractive indexes of oil droplets and water (dispersants) were 1.46 and 1.33, respectively (26).

### Retention Rate of Curcumin

The retention rate of curcumin in HIPEs stabilized by A-BCNs/SPI colloidal particles was measured under accelerated oxidation (27). About 1 g of samples were dissolved in 4 ml chloroform and then centrifuged at 3,000 rpm for 10 min, the absorbance of supernatant was read at 419 nm. The curcumin concentration of samples was calculated by the calibration curve of curcumin standards. The retention rate after emulsion formation and retention rate under accelerated oxidation were calculated by Equations (1) and (2), respectively:

(1) $$\begin{array}{l} \text{Retention rate after emulsion formation }\left(\text{\%}\right)\;=\frac{{\text{c}}_{1}}{{\text{c}}_{2}}\times 100\text{\%} \end{array}$$

(2) $$\begin{array}{l} \text{Retention rate under accelerated oxidation }\left(\text{\%}\right)\;=\frac{{\text{c}}_{3}}{{\text{c}}_{1}}\times 100\text{\%} \end{array}$$

where *c*_1_ represents the initial concentrations of curcumin encapsulated in HIPEs, *c*_2_ represents the initial concentrations of curcumin loaded in oil, and *c*_3_ represent the concentrations of curcumin loaded in samples under accelerated oxidation.

### DPPH Removal Efficiency

About 1 g of samples were mixed with 4 ml ethanol and centrifuged at 3,000 g for 15 min. Then, 1 ml of supernatant was mixed with 3 ml of DPPH solution (1 mM). The mixture was incubated for 30 min in a dark environment. Then, the absorbance was measured with a spectrophotometer (UV-3000, Insmark, Shanghai, China) at 517 nm (28). DPPH removal efficiency was calculated with the following equation.

(3) $$\begin{array}{l} \text{DPPH removal efficiency }\left(\text{\%}\right)\;=\left(1-\frac{\text{A}-{\text{A}}_{1}}{{\text{A}}_{0}}\right)\times 100\text{\%} \end{array}$$

where *A* represents adsorption after incubation, *A*_0_ represents the adsorption of the initial DPPH solution, and *A*_1_ represents the adsorption of the mix solution.

### Lipid Oxidation

The contents of primary lipid oxidation products [lipid hydroperoxide (LH)] and secondary lipid oxidation products [malondialdehyde (MDA)] in oil, cur-oil, HIPEs, and cur-HIPEs were determined under accelerated oxidation (60°C, 10 days) according to the method in a previous study (29).

### *In vitro* Digestion Model

A gastrointestinal tract model was utilized to evaluate the potential gastrointestinal fate of curcumin in a HIPE system (30). The samples were preheated at 37°C and contain the same level of oil.

For the mouth stage, 20 ml of HIPEs was mixed with 20 ml of mucin solution, which was prepared by using PBS with the mucin concentration of 0.03 g/ml. Then, the pH of the whole system was adjusted to 6.8 and incubated in a table concentrator (37°C, 100 rpm) for 10 min.

For the stomach stage, 15 ml of the sample collected from the mouth stage was mixed with 15 ml of simulated gastric fluids (SGFs, 3.2 mg/ml pepsin, 2 mg/ml NaCl), and the pH was adjusted to 2.0. Next, the mixture was incubated in a table concentrator (37°C, 100 rpm) for 120 min.

For the intestine stage, 1.5 ml of simulated intestine fluids (SIFs, 10 mm CaCl_2_ and 150 mm NaCl) and 3.5 ml of bile salt (20 mg/ml) were added into 30 ml samples from the stomach stage for adjusting the pH to 7.0 using 0.1 M NaOH. Then, 2.5 ml of mixed solutions containing 2.4 mg/ml pancreatin and 3.6 mg/ml lipase were added to the system. Finally, the mixture was incubated in a water bath with magnetic stirrers (37°C, 100 rpm) for 120 min.

### The Release of FFAs

The degree of lipolysis was measured by the release of FFAs (31). During the intestinal digestion, NaOH was added into the whole system to neutralize the FFAs. The consumption of NaOH was recorded for every 10 min. The fraction of FFA release was calculated by Equation 4.

(4) $$\begin{array}{l} \text{FFA }\left(\%\right)\;=\frac{V_{\text{NaOH}}\times m_{\text{NaOH}}\times M_{\text{lipid}}}{2\times W_{\text{lipid}}}\times 100\% \end{array}$$

where *V*_NaOH_ is the volume of the NaOH for the neutralization of the FFAs, m_NaOH_ is the concentration of NaOH solutions (0.1 M), *M*_lipid_ is the average molecular mass of oil, and *W*_lipid_ is the total content of oil during the intestinal digestion.

### Bioaccessibility of Curcumin

After intestinal digestion, the samples were collected for the determination of the bioaccessibility of curcumin. The samples were centrifuged at 10,000 *g* for 30 min. Then, the clear micelle phase and raw digesta, which dissolved curcumin, were measured by using HPLC (32). The curcumin concentration of samples was calculated by the calibration curve of curcumin standards. The bioaccessibility of curcumin was calculated by Equation 5.

(5) $$\begin{array}{l} Bioaccessbility\;\left(\%\right)\;=\frac{C_{micelle}}{C_{digest}}\times 100\% \end{array}$$

where *c*_micelle_ is the concentration of curcumin in micelle and *c*_digest_ is the concentration of curcumin in the whole digest.

### Statistical Analysis

Analysis of variance (ANOVA) of the data was performed by using the SPSS 22 (SPSS Inc., Chicago, IL, USA), and the data were expressed as mean values ± SD with a CI of 95%. All data were plotted using the Origin 2019 software (Origin Lab Inc., Northampton, MA, USA).

## Results and Discussion

### Zeta-Potential and Particle Size Distributions

In this study, the particle size distribution and ζ-potential of BCNs, SPI, and BCNs/SPI colloidal particles were analyzed. As described in Table 1, the average size of BCNs/SPI colloidal particles with these two self-assembly methods displayed a significant difference (*p* < 0.05), where the average size of S-BCNs/SPI colloidal particles (1,495.41 ± 248.01 nm) was significantly larger than that of A-BCNs/SPI colloidal particles (1,059.53 ± 104.77 nm). In addition, the polydispersity indexes of complex colloidal particles *via* the anti-solvent precipitation and a simple complex method were 0.27 ± 0.03 and 0.24 ± 0.02, respectively, which were lower than that of BCNs (0.37 ± 0.03) and SPI (0.33 ± 0.02). This result suggests that colloidal particles showed a better distribution in comparison with the single BCNs and SPI. The ζ-potential of SPI and BCNs was −33.07 ± 3.97 and −15.61 ± 0.65 mV, respectively (Table 1). For complex particles, the ζ-potential of A-BCNs/SPI (−35.97 ± 1.54 mV) was significantly higher than that of S-BCNs/SPI colloidal particles (−14.80 ± 1.06 mV), suggesting that the repulsive forces between the particles self-assembled by the anti-solvent method were intensively larger than those between BCNs/SPI particles self-assembled by a simple complex method. Thus, particles with weaker repulsive forces were easy aggregated, thus leading to the larger particle sizes as described earlier. Additionally, the ζ-potential of BCNs/SPI self-assembled by the anti-solvent method was higher than that of both BCNs and SPI, indicating that surface patch binding would participate in the formation of BCNs/SPI colloidal particles (33).

**Table 1:** Average size, polydispersity index (PDI), and zeta- (ζ-) potential of bacterial cellulose nanofibers (BCNs), soy protein isolate (SPI), and BCNs/SPI colloidal particles with different self-assembly mechanisms.

**Types**	**BCNs**	**SPI**	**A-BCNs/SPI**	**S-BCNs/SPI**
Average size (nm)	366.71 ± 45.2^d^	947.24 ± 157.42^c^	1059.53 ± 104.77^b^	1495.41 ± 248.01^a^
PDI	0.37 ± 0.03^a^	0.33 ± 0.02^a^	0.27 ± 0.03^b^	0.24 ± 0.02^b^
Zeta potential (mV)	−15.61 ± 0.65^b^	−33.07 ± 3.97^a^	−35.97 ± 1.54^a^	−14.80 ± 1.06^b^

*Data are presented as means ± SD with three replications. Different letters (a and b) show a significant difference in the same row*.

*^c,d^This indicated a significant difference between the groups*.

### Microstructure of BCNs, SPI, and BCNs/SPI Colloidal Particles

**Figure 1:**
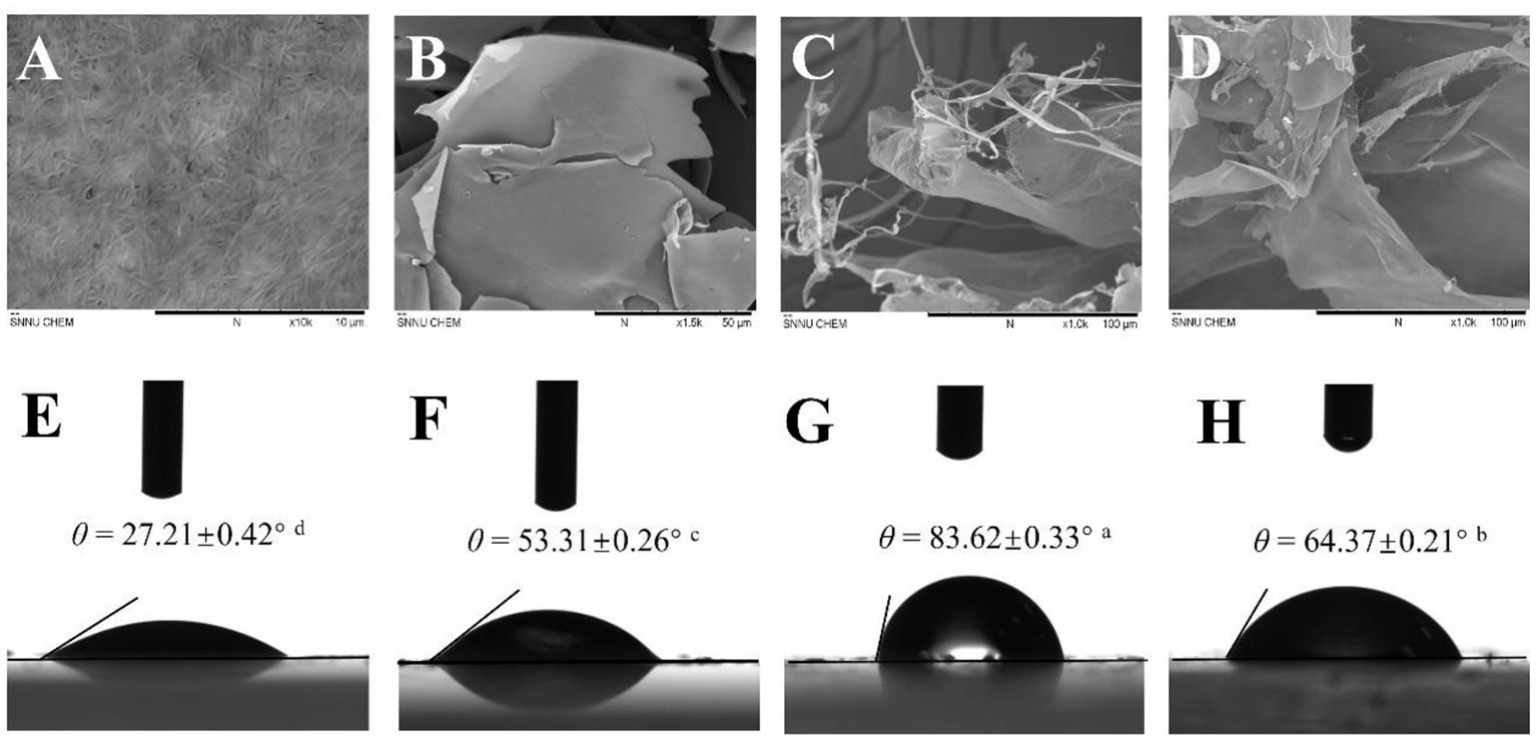
Scanning electron microscopy (SEM) images of bacterial cellulose nanofibers (BCNs) (**A**, at 10.0 K magnification), soy protein isolate (SPI) (**B**, at 1.5 K magnification), A-BCNs/SPI colloidal particles (**C**, at 1.0 K magnification), and S-BCNs/SPI colloidal particles (**D**, at 1.0 K magnification). Water contact angle values of BCNs **(E)**, SPI **(F)**, A-BCNs/SPI colloidal particles **(G)**, and S-BCNs/SPI colloidal particles **(H)**.

The morphologies of BCNs, SPI, and BCNs/SPI colloidal particles with different self-assembly methods were observed by using the SEM. As displayed in Figure 1A, BCNs revealed an irregularly interconnected 3D network, which was in agreement with our results in a previous study (23). The structure of SPI showed a lamellar structure with smooth surfaces as a result of the sample treatment in SEM analysis (Figure 1B). The structure of BCNs/SPI colloidal particles with different self-assembly methods displayed a cross-linked structure with long fibers and an SPI matrix. The structure of BCNs/SPI colloidal particles self-assembled by a simple complex method showed a smooth agglomerate with a less fiber structure (Figure 1D). This phenomenon suggested that BCNs were covered by SPI and there were less structural rearrangements. However, BCNs/SPI colloidal particles self-assembled by the anti-solvent precipitation displayed a rough surface, where a large number of fibers passed through and were distributed in an SPI matrix (Figure 1C).

### Fourier Transform-IR Spectroscopy

**Figure 2:**
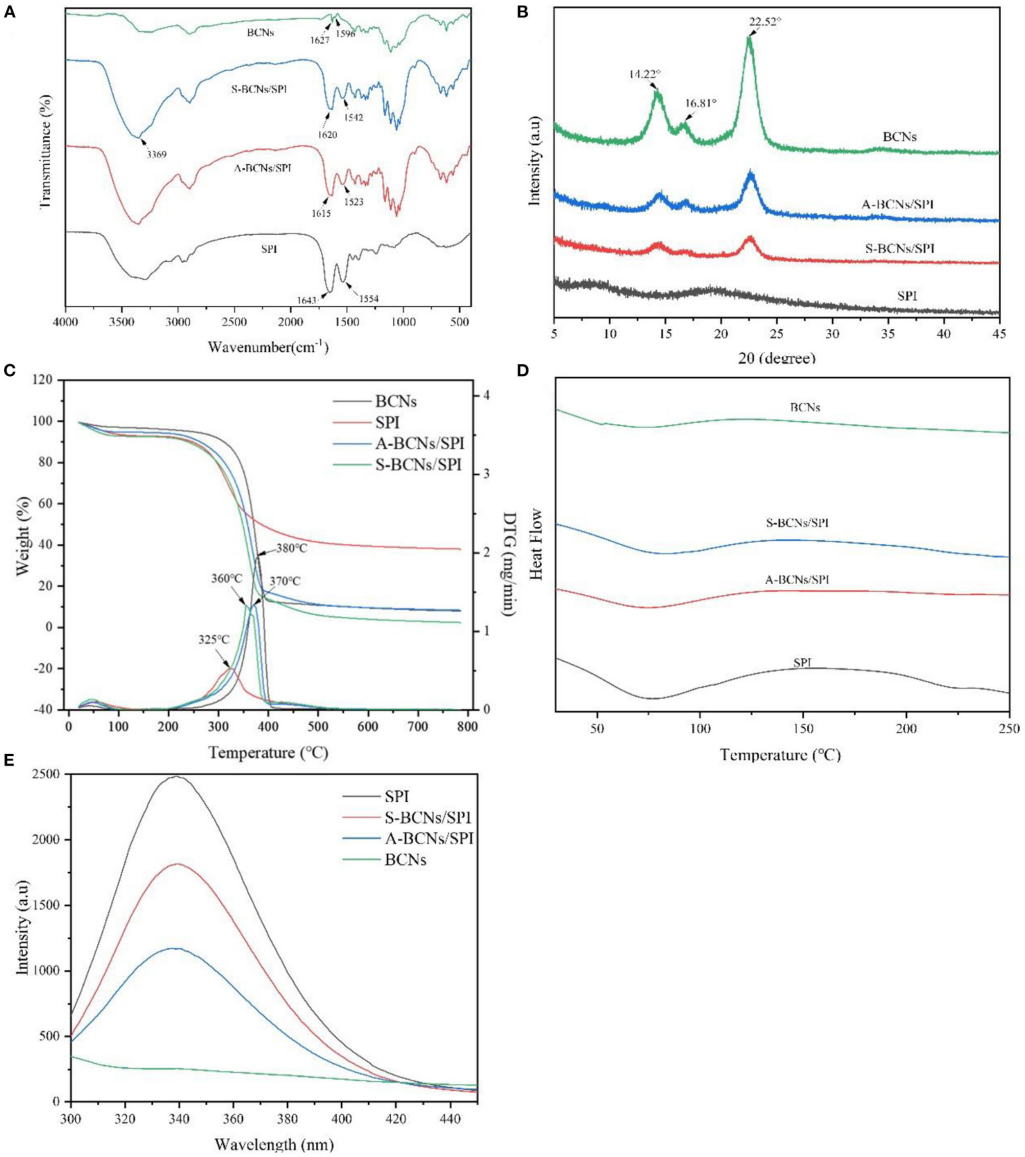
Fourier transform IR (FT-IR) **(A)**, x-ray diffraction (XRD) **(B)**, thermogravimetric analysis (TGA) **(C)**, differential scanning calorimetry (DSC) **(D)**, and fluorescence spectrums **(E)** of SPI, BCNs, and BCNs/SPI colloidal particles prepared by using different self-assembly methods.

In this study, to investigate the changes in the chemical structure of colloidal particles with different self-assembly methods, FT-IR analysis was performed. As shown in Figure 2A, in the spectrum of BCNs, a wide band in the range of 3,600–3,000 cm^−1^ belonged to O-H stretching of hydroxyl groups of BCNs, which was in accordance with the reported result (34). The characteristic peaks at around 2,908 and 1,428 cm^−1^ were associated with the dissymmetric stretching vibration and unsymmetric distortion vibration of methylene (-CH2-). In the spectrum of SPI, the characteristic peak of O-H stretching vibrations exhibited at 3,274 cm^−1^, and obvious absorption peaks at 1,554 and 1,643 cm^−1^ were assigned to the N-H bending vibration (amide II) and C-O stretching vibration (amide I), respectively (35). The absorption peaks of N-H bending vibration (amide II) and C-O stretching vibration (amide I) in BCNs/SPI colloidal particles self-assembled by the anti-solvent precipitation were observed at 1,523 and 1,615 cm^−1^, respectively while the corresponding peaks shifted to higher wavenumbers of 1,542 and 1,620 cm^−1^ in BCNs/SPI colloidal particles with a simple complex method, respectively. This result indicated that hydrogen bonds are generated in BCNs/SPI colloidal particles, which is in agreement with the results reported in the literature (36). In addition, the shift distance of A-BCNs/SPI colloidal particles was larger than that of S-BCNs/SPI colloidal particles, indicating that the strengthening of hydrogen bonds generated in BCNs/SPI colloidal particles self-assembled by the anti-solvent method seemed to be stronger than those generated in BCNs/SPI colloidal particles self-assembled by a simple complex method.

### X-Ray Diffraction

The crystal properties of BCNs, SPI, and BCNs/SPI colloidal particles were investigated *via* an XRD analysis. As displayed in Figure 2B, there were no peaks in the pure SPI, suggesting that amorphous humps were presented in pure SPI, which were in accordance with the reported result (37). There were three characteristic peaks appearing at 2θ = 14.22, 16.81, and 22.52° in the XRD profile of BCNs, suggesting that BCNs possess a typical cellulose I structure with a high crystal structure, which was in agreement with our previous result (38). The crystallinity of BCNs/SPI colloidal particles obtained *via* different methods shows a decreased value in comparison with that of BCNs, which was probably due to the interactions between BCNs and SPI (23). Furthermore, the crystallinity of A-BCNs/SPI colloidal particles was higher than that of S-BCNs/SPI colloidal particles, which was likely because the rough surface of fiber structures was largely covered by the SPI in the particles self-assembled by a simple complex method, confirmed by the SEM results as described earlier.

### Thermostability

The TGA and DTG curves of BCNs, SPI, and BCNs/SPI colloidal particles were shown in Figure 2C. The weight loss stages at relatively low temperatures were associated with the volatilization of the physical and chemical bound water or other volatiles and the dehydration in all samples, and the weight loss was about 1%, which was in agreement with the result reported in the literature (39). With an increase in temperature, the thermograph showed different degradation behaviors between BCNs and BCNs/SPI colloidal particles, where the main degradation of BCNs started at 200°C while the main degradation of BCNs/SPI colloidal particles started at around 150°C, which was attributed to the thermal denaturation of soybean protein components including β-conglycinin and glycinin fractions. Additionally, the main degradation temperature of BCNs/SPI colloidal particles *via* the anti-solvent precipitation was relatively higher than that of BCNs/SPI colloidal particles *via* a simple complex method. Based on the DTG analysis, the main degradation temperature of SPI was 325°C, which was in coincidence with the result reported in the literature (40). The peaks at 360 and 370°C were presented in the curves of BCNs/SPI colloidal particles self-assembled by a simple complex method and the anti-solvent precipitation, respectively. The main degradation temperature of BCNs/SPI colloidal particles *via* different methods increased in comparison with that of SPI, indicating that the thermostability of colloidal particles was improved by BCNs. The present result showed that the temperature of the maximum weight reduction of A-BCNs/SPI colloidal particles was markedly higher than that of S-BCNs/SPI colloidal particles, suggesting that A-BCNs/SPI colloidal particles possessed higher thermal stability in comparison with S-BCNs/SPI colloidal particles. This result was mainly caused by the higher crystallinity of A-BCNs/SPI colloidal particles in comparison with that of S-BCNs/SPI, which was confirmed by the XRD results as described earlier.

The differential scanning calorimetry curve was displayed in Figure 2D. Two endothermic peaks at 63.26 and 134.28°C in BCNs were attributed to the degradation of crystalline water molecules and the degradation of polymers, respectively. A clear endothermic peak at 50–125°C was presented in SPI and complex colloidal particles, which was mainly attributed to β-conglycinin (7S) and glycinin (11S) in SPI (41). This result indicated that the physical interaction between BCNs and SPI was generated during a self-assembly process other than a chemical interaction, which was confirmed by the FT-IR results as described earlier. In addition, with an increase in temperature, a melting peak at 230°C in the DSC curve was observed in SPI and disappeared in the curve of BCNs/SPI colloidal particles, indicating that BCNs improved the thermostability of SPI, which was in consistent with the TGA profile as described earlier.

### Contact Angle

The interfacial wettability of colloidal particles is an important property for evaluating the emulsifying capacity of colloidal particles as Pickering stabilizers (42). Contact angle measurement is a direct method for the characterization of the partial wettability of a solid particle. As shown in Figure 1, the contact angle values of BCNs and SPI were 27.21° ± 0.42° and 53.31° ± 0.26°, suggesting strong hydrophilicity as a result of the hydroxyl groups of BCNs and SPI, which is in agreement with the FT-IR results as described earlier. Moreover, the contact angle values of colloidal particles were higher than those of both BCNs and SPI. This result was probably due to the hidden and closed surface hydrophobicity groups of SPI getting exposed during the self-assembly process as a result of the presence of hydrogen bonds between the hydroxyl groups of BCNs and carbonyl groups of SPI (43). It has been demonstrated that θ value is much closer to 90°, the wettability of particles facilitates more effective adsorption and accumulation at the droplet surface, which is more suitable for the preparation of stable Pickering emulsion (1). The contact angle of A-BCNs/SPI colloidal particles (83.62° ± 0.33°) was higher than that of S-BCNs/SPI colloidal particles (64.37° ± 0.20°), suggesting that A-BCNs/SPI colloidal particles displayed a better amphiphilic property as a result of their contact angle values closer to 90°. Thus, the emulsifying capacity of A-BCNs/SPI colloidal particles was higher than that of S-BCNs/SPI colloidal particles.

### Fluorescence Property

In the present study, the conformational changes of proteins were determined using fluorescence spectroscopy. As shown in Figure 2E, the fluorescence intensity of BCNs was much lower than that of SPI, which was likely due to the non-protein structure of BCNs. SPI presented a typical fluorescence emission peak at 340 nm, which was in agreement with a previous study (44). The wavelength of maximum emission did not show a distinct shift in both two types of colloid particles while the fluorescence intensity of colloid particles obviously (*p* < 0.05) decreased as a result of the presence of BCNs, which was in agreement with the reported result that the protein conformation was altered as a result of the interaction with other biopolymers (45). Moreover, the intensity of A-BCNs/SPI colloidal particles was significantly lower than that of S-BCNs/SPI colloidal particles, indicating that the effect of the anti-solvent precipitation on the protein conformation was more dramatically in comparison with that of a simple complex method.

### Rheological Properties of BCNs/SPI Colloidal Particles

**Figure 3:**
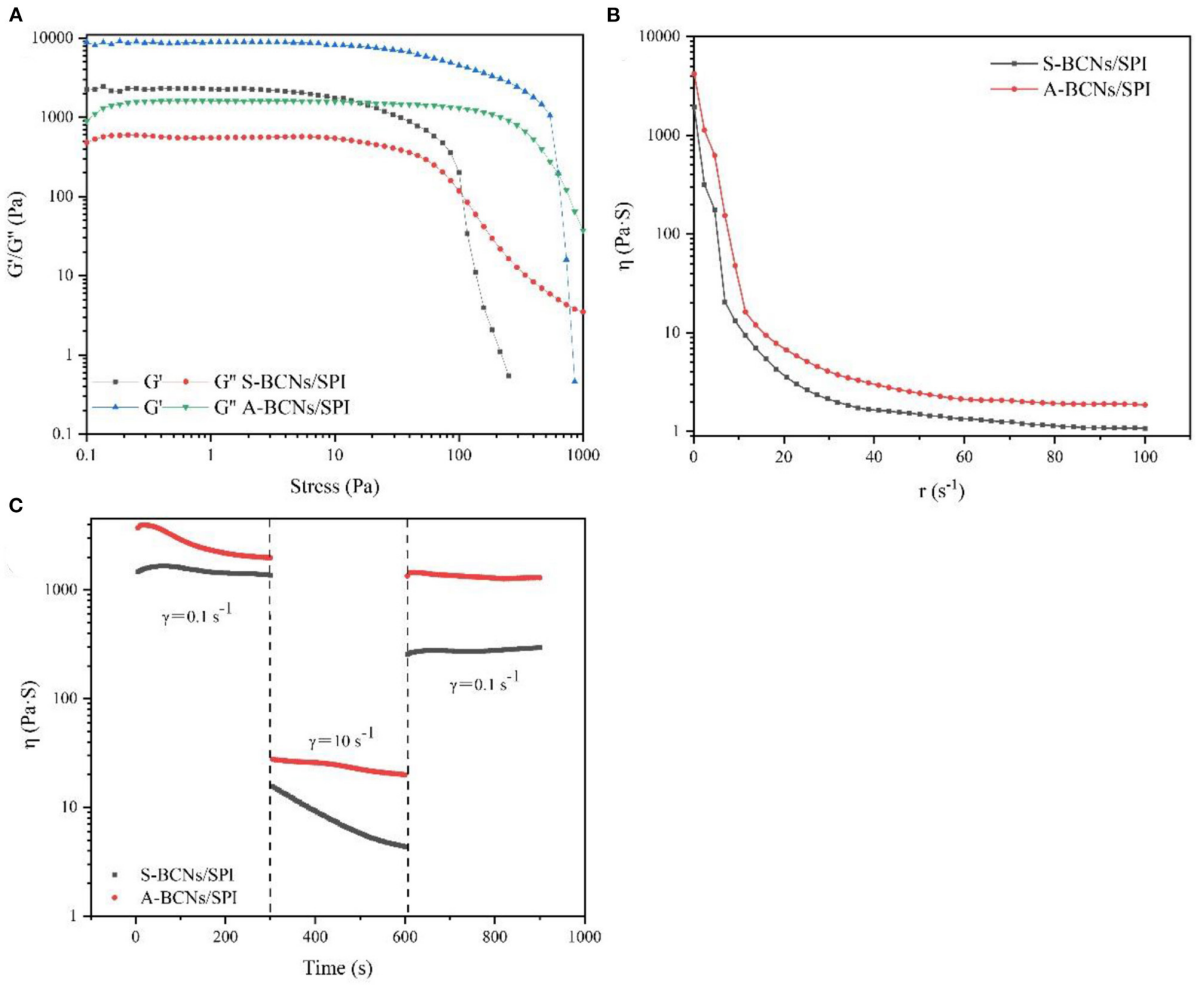
Stress sweeps **(A)**, apparent viscosity **(B)**, and thixotropic recovery **(C)** for BCNs/SPI colloidal particles prepared by using different self-assembly methods.

In this study, the rheological behavior of BCNs/SPI colloidal particles *via* different methods was investigated. As shown in Figure 3A, colloidal particles exhibit a storage modulus (G′), which is higher than a loss modulus (G″), indicating that all particles possessed gel-like behaviors of elastic solids, which was in agreement with the reported result (46). The G′ of BCNs/SPI colloidal particles self-assembled by the anti-solvent precipitation was higher than that of the colloidal particles self-assembled by a simple complex method at the linear viscoelastic interval, suggesting that the colloidal particles self-assembled by the anti-solvent precipitation had stronger gel viscoelasticity. With an increase in stress, an obvious intersection (yield point) appeared, which indicated the changes of gel viscoelasticity, showing the conversion from gel-like behaviors to sol-like behaviors (47). The corresponding crossover point increased from 106 Pa for S-BCNs/SPI colloidal particles to 958 Pa for A-BCNs/SPI colloidal particles. These results suggested that the texture of colloidal particles using the anti-solvent precipitation was stronger than that of colloidal particles self-assembled using a simple complex method.

Figure 3B describes the dynamical viscosity (η) of colloidal particles as a function of shear rate (d*r*/d*t*) at 25°C. It was observed that the apparent viscosity of all colloidal particles displayed a constant decrease with an increase of shear rate at a relatively lower shear rate (0–50 s^−1^), showing a shear-thinning behavior, suggesting that BCNs/SPI colloidal particles belonged to the category of Newtonian fluid at a relatively lower shear rate (48). However, the apparent viscosity of all colloidal particles displayed stable and unchangeable values at a higher shear rate (60–100 s^−1^), in accordance with the Non-Newton fluidity. In addition, the viscosity of S-BCNs/SPI colloidal particles was lower than that of A-BCNs/SPI colloidal particles, indicating that BCNs/SPI colloidal particles self-assembled by the anti-solvent precipitation showed stronger gel strength, in agreement with the results of the stress sweep curve as described earlier.

The structural recovery properties of both two types of colloidal particles were shown in Figure 3C. The viscosity of A-BCNs/SPI colloidal particles was significantly higher than that of S-BCNs/SPI colloidal particles at all time intervals, which was in agreement with the dynamic viscosity as described earlier. The structural recovery degree was evaluated using the value of maximum viscosity at a third interval divided by the end viscosity of the first interval. The structural recovery degree of A-BCNs/SPI colloidal particles and S-BCNs/SPI colloidal particles was 98.47 and 30.69%, respectively. According to a previous study, the recovery percentage larger than 70% was regarded as a better thixotropic recovery (49). Thus, the result indicated that the structural recovery property of A-BCNs/SPI colloidal particles was better than that of S-BCNs/SPI colloidal particles, which was mainly due to a stronger gel structure demonstrated in the stress sweep as described earlier.

To better understand the underlying mechanism of BCNs/SPI colloidal particles with different self-assembly methods, we proposed a schematic illustration to explain the formation route of BCNs/SPI colloidal particles with different self-assembly methods (Supplementary Figure 2).

### Properties of HIPEs Stabilized by the Prepared Two Types of Colloidal Particles

**Figure 4:**
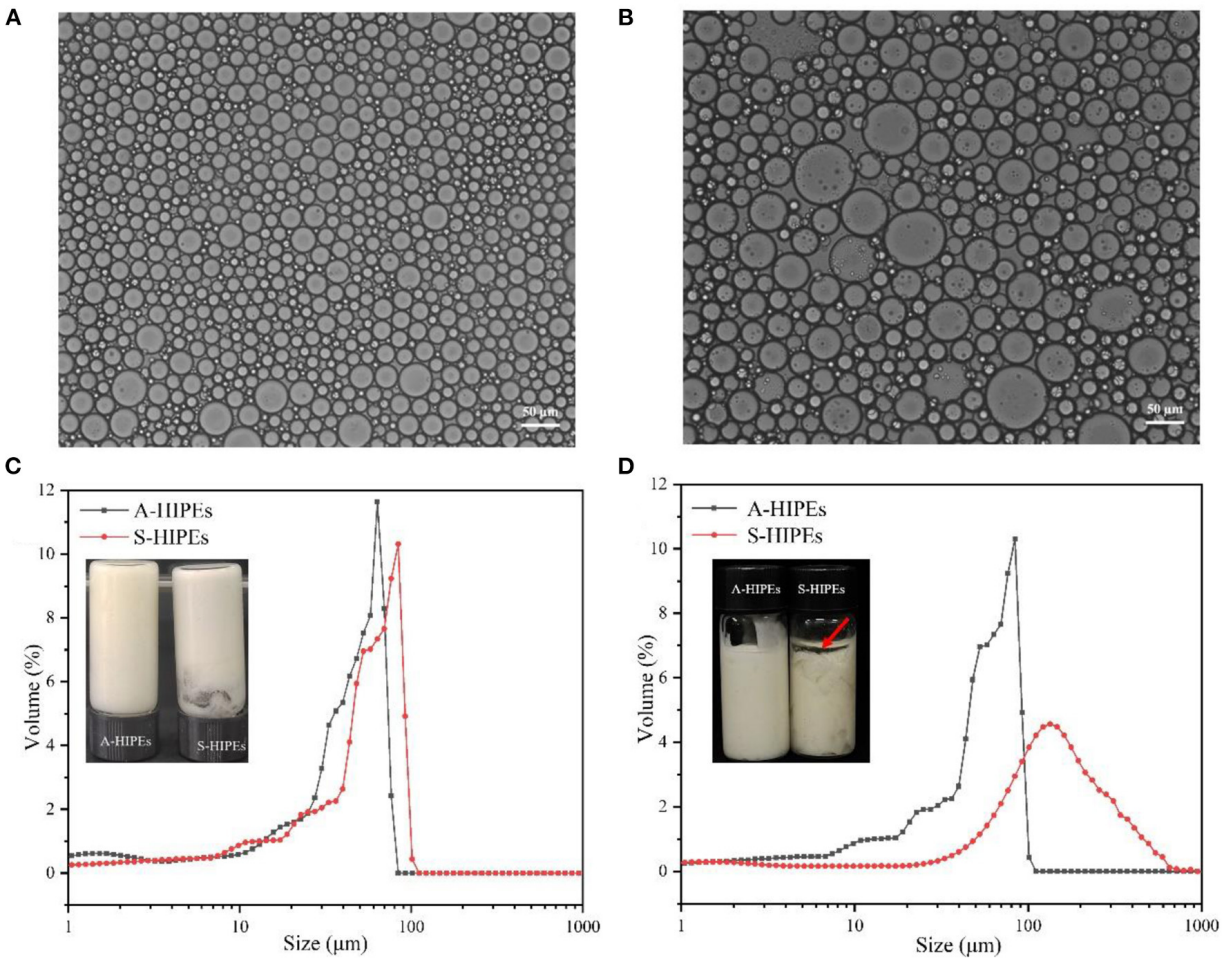
Optical micrographs of A-HIPEs **(A)** and S-HIPEs **(B)**. The bar lengths of optical micrographs represented 50 μm. The appearance and size distribution of the HIPEs stabilized by BCNs/SPI colloidal particles using different self-assembly methods (**C**: new preparation; **D**: 1 month later).

In the present work, the properties of HIPEs stabilized by the prepared BCNs/SPI colloidal particles with different self-assembly methods were investigated. The microstructures of HIPEs stabilized by different types of BCNs/SPI colloidal particles were observed by using an optical microscope. As shown in Figure 4, the droplets were interconnected and closely arranged. This is basically consistent with the fact that a gel-like network for HIPEs would be formed (50). It was obvious that HIPEs stabilized by A-BCNs/SPI colloidal particles presented a small and uniform spherical droplet, whereas HIPEs stabilized by S-BCNs/SPI colloidal particles presented uneven spheres. This result was mainly due to a stronger steric force and the surface activity of the A-BCNs/SPI colloidal particles.

The droplet size distribution of these two types of HIPEs was shown in Figure 4C, it was observed that both two types of HIPEs showed a single peak, whereas the average sizes of these two types of HIPEs displayed a significant difference, where the average size of HIPEs stabilized by A-BCNs/SPI colloidal particles (40.54 ± 2.66 μm) was lower than that of HIPEs stabilized by S-BCNs/SPI colloidal particles (73.96 ± 8.43 μm). Furthermore, the droplet distribution and average size of HIPEs stabilized by A-BCNs/SPI colloidal particles displayed no significant change after 1 month of storage at room temperature, suggesting that HIPEs stabilized by A-BCNs/SPI colloidal particles were very stable. However, there were dramatic changes in the droplet distribution and average size for HIPEs stabilized by S-BCNs/SPI colloidal particles, where the average size of HIPEs stabilized by S-BCNs/SPI colloidal particles significantly increased after 1 month of storage. Moreover, HIPEs stabilized by S-BCNs/SPI colloidal particles seemed to show an oil layer on the top of HIPEs, denoted by a red arrow (Figure 4D). This result suggested that HIPEs stabilized by A-BCNs/SPI colloidal particles were more stable in comparison with those stabilized by S-BCNs/SPI colloidal particles.

The ζ- potential of HIPEs was shown in Table 2. Both these types of HIPEs seemed to possess a negative charge, which was mainly due to the colloidal particles with a negative charge in an oil–water interface. The ζ-potential of HIPEs stabilized by A-BCNs/SPI colloidal particles and S-BCNs/SPI colloidal particles were −35.97 ± 1.54 and −24.30 ± 3.84 mV, respectively. It has been reported that the absolute value of ζ-potential >30 can confer stability in which the emulsion system will resist aggregation (51). In this regard, HIPEs stabilized by A-BCNs/SPI colloidal particles showed better stability than those stabilized by S-BCNs/SPI colloidal particles, which were consistent with the results of the droplet distribution and long-term storage test as described earlier.

**Table 2:** Average size and ζ-potential of A-HIPEs and S-HIPEs.

**Types**	**S-HIPEs**	**A-HIPEs**
Average size (μm)	73.96 ± 8.43^a^	40.54 ± 2.66^b^
Zeta potential (mV)	−24.30 ± 3.84^b^	−35.97 ± 1.54^a^

*Data are presented as means ± SD with three replications. Different letters (a and b) show a significant difference in the same row*.

**Figure 5:**
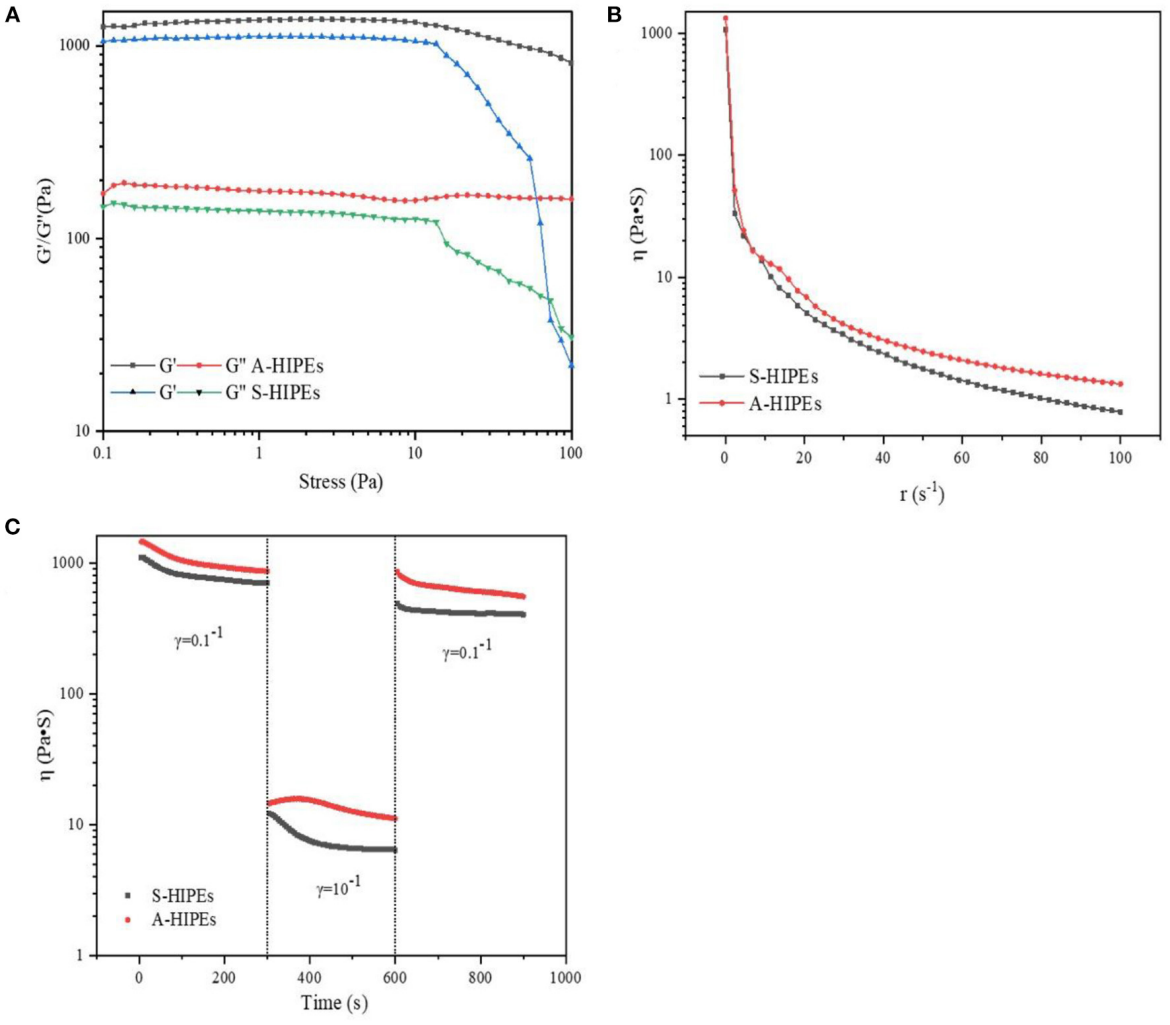
Stress sweeps **(A)**, apparent viscosity **(B)**, and thixotropic recovery **(C)** for HIPEs stabilized by BCNs/SPI colloidal particles with different self-assembly methods.

To better understand the internal structures and physicochemical properties of these emulsions, in the present study, the rheological properties of HIPEs stabilized by BCNs/SPI colloidal particles with different self-assembling methods were investigated. Figure 5 showed the storage modulus (G′), loss modulus (G″), viscosity, and structural recovery properties of HIPEs stabilized by different types of colloidal particles. HIPEs stabilized by different types of colloidal particles showed a storage modulus (G′), which was higher than a loss modulus (G″) in a linear viscoelastic domain while a crossover point at 75.65 Pa was observed in the curve of HIPEs stabilized by S-BCNs/SPI colloidal particles, indicating a weaker structure formed by the S-BCNs/SPI colloidal particles at an interface (5). The stress sweep of HIPEs demonstrated that HIPEs stabilized by A-BCNs/SPI colloidal particles presented better gel viscoelasticity in comparison with the HIPEs stabilized by S-BCNs/SPI colloidal particles. This result was mainly due to an efficient rearrangement of A-BCNs/SPI colloidal particles at an interface into a more ordered network (52). The viscosity test showed that two types of HIPEs presented a characteristic of shear thinning, which was probably due to the squeezing between droplets, leading to a sharp shrink of the continuous phase, in agreement with the result in previous research (1). Additionally, HIPEs stabilized by A-BCNs/SPI colloidal particles displayed a higher viscoelasticity than those stabilized by S-BCNs/SPI colloidal particles, suggesting that the structural recovery degree of HIPEs stabilized by A-BCNs/SPI colloidal particles (99.86%) was significantly higher than that of HIPEs stabilized by S-BCNs/SPI colloidal particles (78.51%). This result was mainly due to improved gel viscoelasticity of HIPEs stabilized by A-BCNs/SPI confirmed by the stress sweep and viscosity test as described earlier.

### Loading Properties of Curcumin in Different Systems

**Figure 6:**
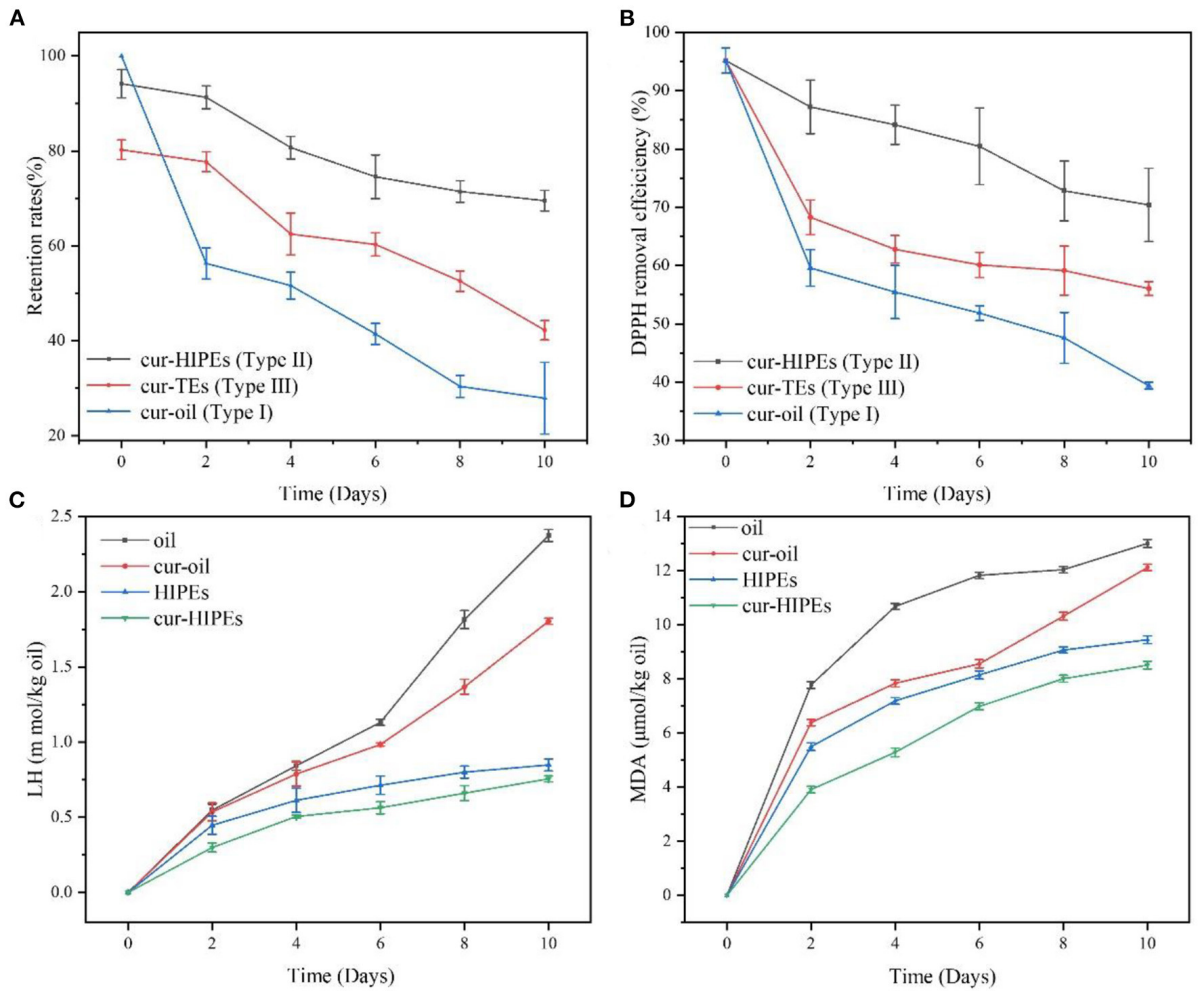
Evolution of lipid hydroperoxides (LHs) **(A)**, malondialdehyde (MDA) **(B)** retention rate **(C)**, and 1,1-diphenyl-2-picrylhydrazyl (DPPH) removal efficiency **(D)** under accelerated storages at 60°C for 10 days.

In this study, three types of systems (Type I: curcumin was loaded with only bulk oil; Type II: curcumin was loaded with HIPEs stabilized by BCNs/SPI colloidal particles *via* the anti-solvent precipitation; and Type III: curcumin was loaded with emulsions stabilized by Tween-80 solution) were used for the loading of curcumin, and the properties of curcumin-loaded systems were investigated. The encapsulated efficiency and retention rate of curcumin in the three systems were evaluated and compared. As shown in Figure 6A, the retention rate of curcumin after the emulsion formation in Type II and Type III systems was 94.18 ± 2.97 and 80.25 ± 2.04%, respectively. After a 10-day storage, the retention rate of curcumin in Type II system showed the highest (69.51 ± 2.14%), followed by that in Type III system (42.22 ± 2.04%), whereas the retention rate of curcumin in Type I system was the lowest (27.90 ± 7.57%). These results suggested that HIPEs stabilized by BCNs/SPI colloidal particles *via* the anti-solvent precipitation displayed better protection for curcumin in comparison with the emulsion stabilized by Tween-80 and bulk oil.

DPPH removal efficiency was evaluated under accelerated oxidation. As shown in Figure 6B, the initial DPPH removal efficiency of all samples was very similar. However, after 10 days of storage, the DPPH removal efficiency of curcumin in Type I system decreased to 39.39 ± 0.58% from 95.15 ± 2.17%. The DPPH removal efficiency of curcumin in Type II system was the highest (70.40 ± 6.28%), followed by that in Type III system (56.15 ± 1.19%), which indicated that the antioxidant ability of curcumin was obviously improved using the system of HIPEs stabilized by BCNs/SPI colloidal particles *via* the anti-solvent precipitation.

### Lipid Oxidation

To better understand the change in the antioxidant ability of curcumin-loaded HIPEs stabilized by BCNs/SPI colloidal particles *via* the anti-solvent precipitation under accelerated oxidation, the contents of lipid oxidation markers (LH and MDA) in the samples were detected. As shown in Figure 6, LH and MDA contents of bulk oil and cur-oil were significantly increased after the storage for 10 days at 60°C. It was obvious that the antioxidant effects of HIPEs systems were significantly better than those of a bulk oil system, which was mainly attributed to the formation of a solid physical barrier at an oil/water interface because of the irreversible adsorption of BCNs/SPI colloidal particles *via* the anti-solvent precipitation (52). Furthermore, the barriers would protect the oil from oxygen, thus improving the antioxidant effect of the HIPE system. LH and MDA contents of curcumin-loaded oil and curcumin-loaded HIPEs were obviously lower than the bulk oil and HIPEs during the storage, respectively, suggesting that the curcumin decreased the lipid oxidation rate in agreement with the previous result as reported in the literature (5).

### Digest Properties of Curcumin in Different Systems

**Figure 7:**
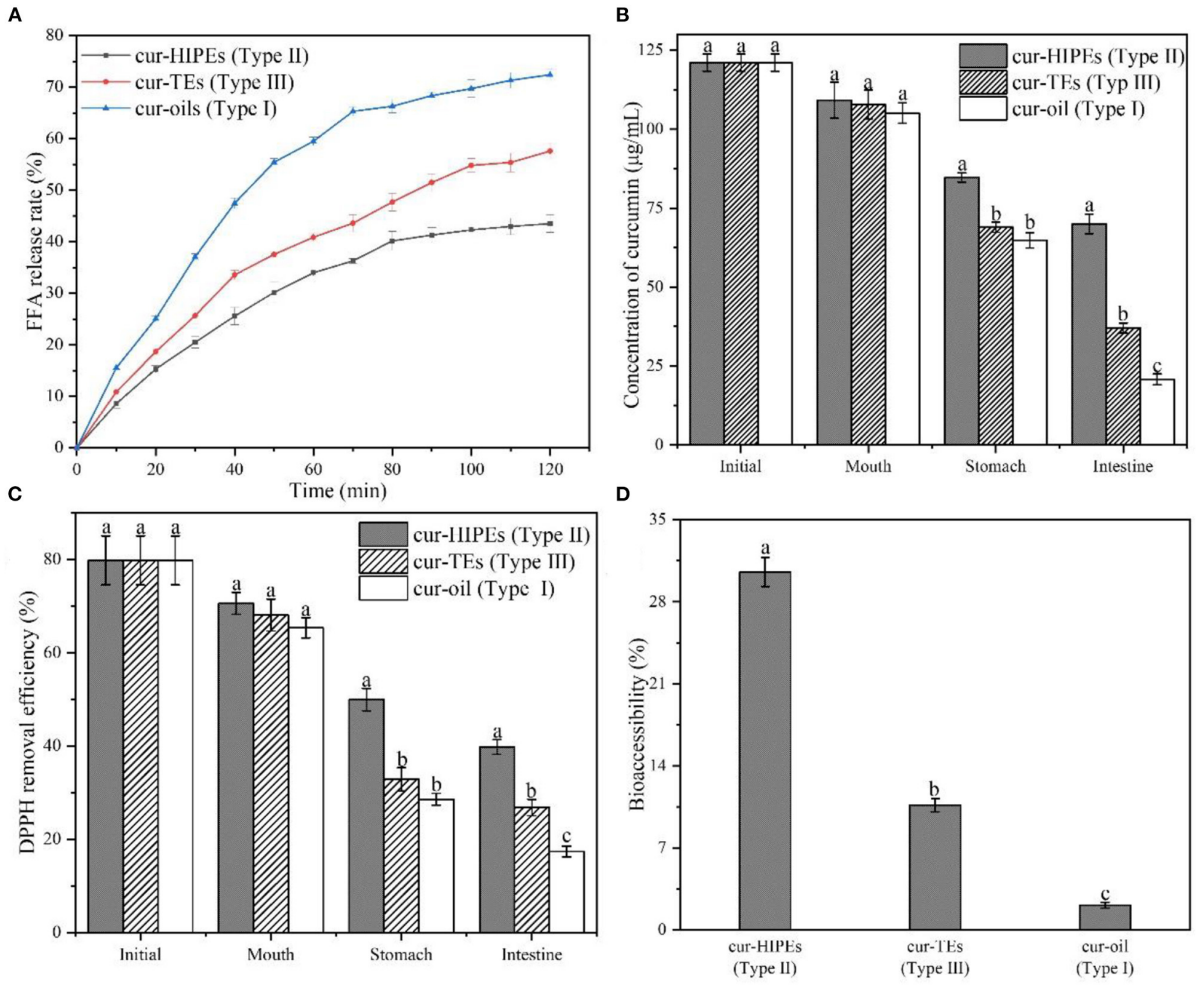
FFA release rate **(A)**, concentration of curcumin **(B)**, 1,1-diphenyl-2-picrylhydrazyl (DPPH) removal efficiency **(C)**, and bioaccessibility of curcumin **(D)** during *in vitro* digestion.

In the present work, three types of systems (Type I: curcumin was loaded with only bulk oil; Type II: curcumin was loaded with HIPEs stabilized by BCNs/SPI colloidal particles *via* the anti-solvent precipitation; and Type III: curcumin was loaded with emulsions stabilized by Tween-80 solution) were used for the delivery of curcumin, and the *in vitro* digestion properties of curcumin-loaded systems were investigated. Figure 7A shows the FFA release profiles of curcumin in the three types of systems. For all systems, an increase in the FFA release rate was observed during the simulated gastric digestion, which was due to the conversion of triglyceride into FFAs and glycerides by the absorption of lipase on the surface of an oil droplet in agreement with the previous research (31). Type I system showed the highest FFA release rate (72.40 ± 1.05%) while the FFA release rate of Type II system was the lowest (43.52 ± 1.71%), followed by that of Type III system being 57.58 ± 1.49%. This phenomenon was mainly attributed to the formation of a solid physical barrier in the HIPE system formed by BCNs/SPI colloidal particles because it has been demonstrated that the solid physical barriers were hardly disrupted by surface active bile salts and lipase due to their irreversible adsorption at an oil/water interface (53). In contrast, the adsorption of Tween-80 at an oil/water interface was unstable, and the structure of the emulsion was destroyed during gastric digestion. In contrast, the adsorption of Tween-80 at an oil/water interface was unstable, thus the structure of emulsion was easily disrupted during *in vitro* digestion.

The concentrations of curcumin in different systems during *in vitro* digestion were determined by using HPLC. As shown in Figure 7B, the initial curcumin concentration of all systems was similar (121.06 ± 2.71 μg/ml). After *in vitro* digestion, the concentration of curcumin in Type II system was the highest with a value of 69.95 ± 3.14 μg/ml whereas the concentration of curcumin in Type I and Type III systems was 20.73 ± 1.71 and 37.01 ± 1.58 μg/ml, respectively. Thus, after digestion, the retention rate of curcumin in Type II system was the highest (57.78%) while that of curcumin in Type I and Type III systems was 17.12% and 30.57%, respectively. This result indicated that the HIPEs potentially protect the curcumin from getting destroyed from the simulated digestion fluids during *in vitro* digestion. To investigate the change in the antioxidant ability of curcumin during *in vitro* digestion, DPPH removal efficiency was evaluated. As shown in Figure 7C, the DPPH removal efficiency of all systems was 79.81% ± 5.25%. After *in vitro* digestion, the DPPH removal efficiency of curcumin in Type I system was decreased to 17.40% ± 1.15%, whereas the DPPH removal efficiencies of curcumin in Type II and Type III systems were 39.81% ± 1.58% and 26.86% ± 1.75%, respectively. This result suggested that the structure of HIPEs increased the DPPH removal efficiencies of curcumin during *in vitro* digestion, in agreement with the change in curcumin concentration during *in vitro* digestion as shown in Figure 7C.

The bioaccessibility of curcumin in different systems during *in vitro* digestion was shown in Figure 7D, the bioaccessibility of curcumin in Type I, Type II, and Type III systems was 2.10% ± 0.25, 30.54% ± 1.25, and 10.65% ± 0.56%, respectively. The bioaccessibility of curcumin in Type II system was higher than that of curcumin in Type I and Type III systems. This result was probably obtained by HIPEs having the ability to improve the solubility of curcumin in micelle after *in vitro* digestion. In the HIPE system, the undigested BCNs/SPI colloidal particles may improve the solubility of curcumin in mix micelle (54). In contrast, the solubility of curcumin in mix micelle from Type I and Type III systems was relatively lower, thus leading to the reduced bioaccessibility for curcumin.

## Conclusion

In this study, BCNs/SPI colloidal particles were prepared by using the anti-solvent precipitation and a simple complex method. The ζ-potential of colloidal particles indicated that the surface patch binding gets induced during the self-assembly of BCNs and SPI. FT-IR analysis showed that BCNs/SPI colloidal particles were assembled by using the anti-solvent precipitation and a simple complex method through hydrogen bonds. Moreover, the intensity of hydrogen bonds generated in BCNs/SPI colloidal particles self-assembled by the anti-solvent precipitation was stronger than those particles self-assembled by a simple complex method. Meanwhile, the droplet size, crystallinity, thermal stability, and contact angle values of composite colloidal particles self-assembled by the anti-solvent precipitation were better than the colloidal particles self-assembled by a simple complex method. Dynamic oscillatory measurements of colloidal particles indicated that the colloidal particles self-assembled by the anti-solvent precipitation have enhanced gel viscoelasticity. The microstructures and long-term stabilization test displayed that HIPEs stabilized by A-BCNs/SPI particles were more stable as compared to those stabilized by S-BCNs/SPI particles. Curcumin-loaded HIPEs stabilized by BCNs/SPI colloidal particles showed better loading effects and antioxidant ability as compared to the oil system and emulsion system stabilized by Tween-80 under accelerated oxidation. Moreover, the bioaccessibility of curcumin in the HIPE system during *in vitro* digestion was higher as compared to that of curcumin in the emulsion system stabilized by Tween-80. Therefore, it can be concluded that the anti-solvent precipitation is an effective way to assemble the polysaccharide/protein particles that are used as high internal phase Pickering stabilizers, and HIPEs stabilized by A-BCNs/SPI showed a potential for the delivery of curcumin.

## Data Availability Statement

The original contributions presented in the study are included in the article/Supplementary Material, further inquiries can be directed to the corresponding author/s.

## Author Contributions

RS and DL conceived and designed the experiments. RS and ZL performed the experiments. XY and HZ contributed helpful discussion during the experiment. RS and DL analyzed the data and wrote the manuscript. DL, HZ, and XY reviewed and revised the manuscript. All authors contributed to the article and approved the submitted version.

## Funding

This study was supported by the National Natural Science Foundation, China (Grant No. C31701662); the Shaanxi Province Agricultural Science and Technology Innovation and Key Project (2021NY-175); the Key Projects of Central Universities, China (Grant No. GK201902012); and the Supporting Program for Youth Talent, China (20190206).

## Conflict of Interest

The authors declare that the research was conducted in the absence of any commercial or financial relationships that could be construed as a potential conflict of interest.

## Publisher's Note

All claims expressed in this article are solely those of the authors and do not necessarily represent those of their affiliated organizations, or those of the publisher, the editors and the reviewers. Any product that may be evaluated in this article, or claim that may be made by its manufacturer, is not guaranteed or endorsed by the publisher.
