# Supplementary material for: Fabrication of Bacterial Cellulose Nanofibers/Soy Protein Isolate Colloidal Particles for the Stabilization of High Internal Phase Pickering Emulsions by Anti-solvent Precipitation and Their Application in the Delivery of Curcumin
Source: Front Nutr. 2021 Sep 7;8:734620. doi: 10.3389/fnut.2021.734620 (PMC8454892; doi:10.3389/fnut.2021.734620)
Supplement: Supplementary file 1 [file Data_Sheet_1.docx]

**Supporting information**

**
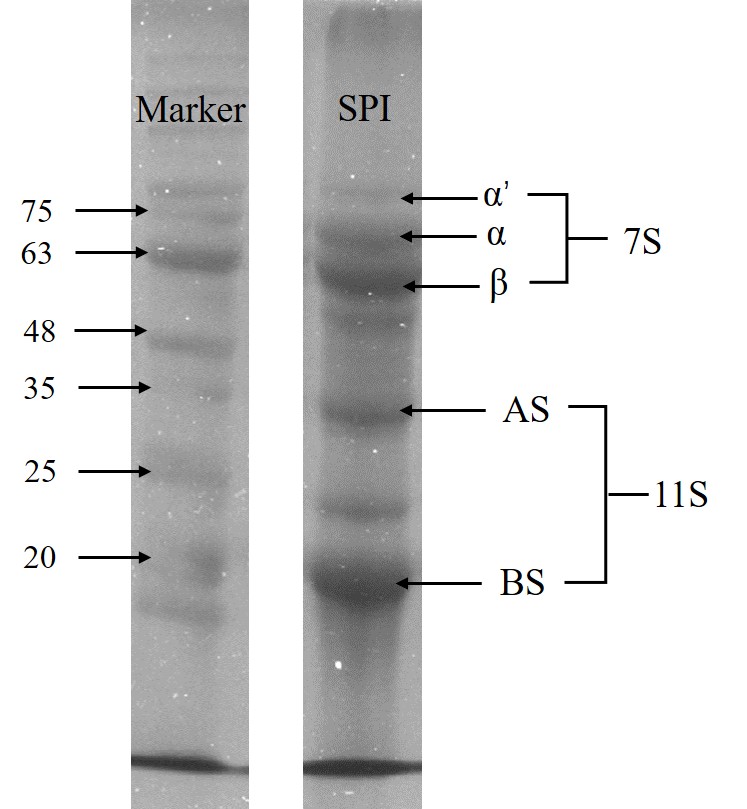
FIGURE. S1.** SDS-PAGE analysis of soy protein isolate
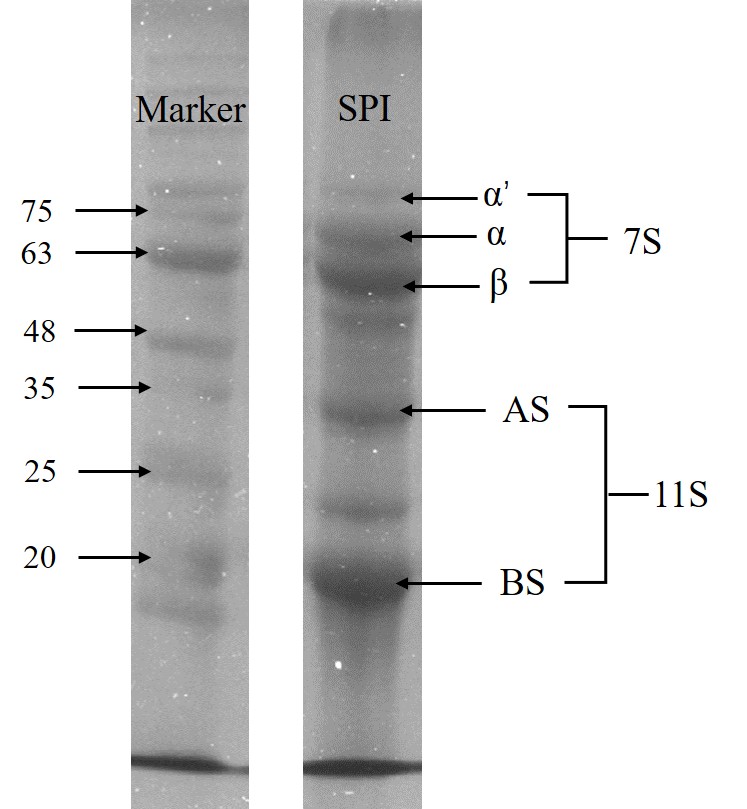
.


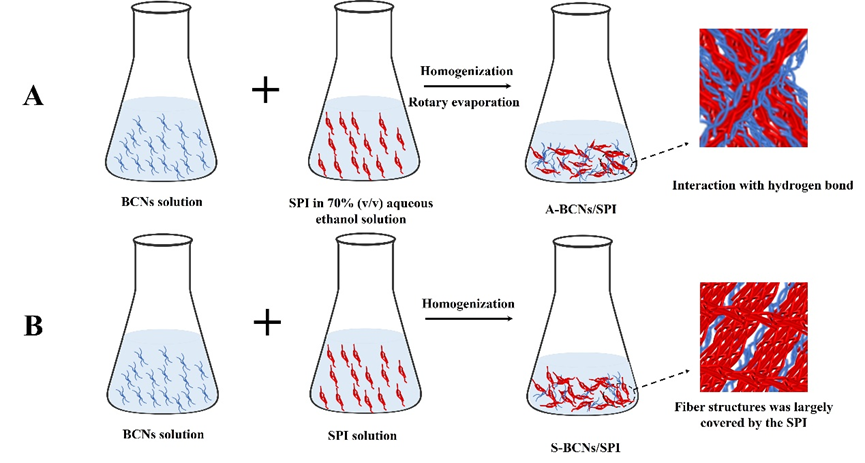


**FIGURE. S2** Schematic mechanism of the BCNs/SPI colloidal particles self-assembled by anti-solvent method (**A**) and simple complex method (**B**).
